# The Hospital Nursing Supplement

**Published:** 1896-03-14

**Authors:** 


"The Hospital" Nursing Mirror.

Being the Extra Nursing Supplement of "The Hospital" Newspaper.

[Contributions for this Supplement should be addressed to the Editor, The Hospital, 428, Strand, London, W.C., and should have the word "Nursing" plainly written in left-hand top corner of the envelope.]

### News from the Nursing World.

### H.R.H. PRINCESS MAUD'S WEDDING PRESENT.

In accordance with the wish of H.R.H. the President of the Royal National Pension Fund, a deputation of nurses attended at Marlborough House on Thursday, 12th inst., to present to the Princess Maud a wedding gift on behalf of upwards of one thousand nurses of the Pension Fund. The deputation, which consisted of twelve nurses (the number arranged by H.R.H.), was made as representative as possible. Invitations were sent to donors connected with metropolitan and provincial general and special hospitals, cottage hospitals, nursing institutions, poor law infirmaries, and private nurses. Ultimately nurses representing the following institutions accepted invitations to assist at the presentation: Guy's, St. George's, St. Thomas's, and the Westminster Hospitals; the Grosvenor Hospital for Women, Lewisham Poor-Law Infirmary, Addenbrooke's Hospital (Cambridge), Royal Portsmouth Hospital, the Home Hospitals' Association (Fitzroy Square), Newcastle Nursing Home, Staffordshire Institution for Nurses, and the Nurses' Co-operation. Nurse Henrietta M. Walker, as an annuitant of the Pension Fund, and one of the first who joined it, was selected to represent the older nurses. We hope to give a full account of the interesting ceremony next week.

### A GENEROUS GIFT.

An offer of generous and timely help has been received by the managers of the Malmesbury Nursing Institute and Cottage Hospital. The accommodation of the latter has for some time been quite inadequate to cope with the increasing needs of the district, but little hope had been entertained of the possibility of raising sufficient money to make the necessary additions yet awhile. Now, however, Colonel Luce, at whose expense the existing cottage hospital was rebuilt in days past, has offered the site at present occupied by two disused houses, and further promised a sum of £500 towards the formation of a building fund. Colonel Luce has also placed at the disposal of the committee the building material contained in the empty houses already on the ground. The impetus thus given by one public-spirited man ought to result in the prompt raising in the neighbourhood of a sufficient sum to enable a hospital to be built which shall be in every respect adequate to the requirements of the district.

### ROYAL PORTSMOUTH HOSPITAL.

The example set at Edinburgh by the election of a lady to serve upon the hospital board of management has been promptly followed at Portsmouth by the election of four women to seats upon the Committee of Management and two upon the House Committee of the Royal Hospital in that borough. The resolutions enabling this to be done were moved by the Rev. W. Hawksley, vicar of All Saints, Portsea, who some short while ago resigned the post of honorary chaplain to the institution in consequence of the absolute refusal of the Committee of Management to allow lady visitors to penetrate within the lock wards. The annual meeting of subscribers, at which, last week, the question came up for discussion, was a distinctly stormy one, an amendment moved by one of the honorary physicians being lost by a minority of one only. Ultimately, however, the resolution was carried, and Miss Agnes Weston, whose work amongst sailors is of world-wide renown, Mrs. Ward, a member of the Board of Guardians, Mrs. Will Hawksley, and Mrs. Guise-Tucker were chosen to occupy the positions. The victory was hardly won, for nearly every member of the existing committee voted against the admission of ladies to its ranks. The result of the experiment, which it is to be hoped may be successful, will be watched with interest by all who concern themselves with hospital management.

### GOOD SERVICE RECOGNISED.

"Very excellent work is done in Dumfries by Nurse Machin." So ran the last report given by Miss Peter of the Dumfries District Nurse; and, at the recent annual meeting of the Dumfries and Maxwelltown Branch of the Queen Victoria's Jubilee Institute for Nurses, very cordial recognition was accorded to Nurse Machin for her faithful work among the sick poor of her district. She was now well known as she went up and down their streets and closes, said the treasurer, "and few people were followed with more grateful affection than she was. The committee felt sure that the subscriptions given so liberally would be even more ungrudgingly offered could their friends see what the work of the district nurse really was in the homes of the poor, how dirt and disorder disappeared, at least temporarily; how tired faces brightened at her entrance, and suffering frames found rest and comfort through her kindly ministrations."

### A WISE POOR LAW INSPECTOR.

It is to be hoped that the St. Asaph Board of Guardians will make haste to follow the sensible counsels of Mr. Bircham, Poor Law Inspector for Wales, delivered at a recent meeting of the Board. An application had been made to the Guardians to subscribe to the District Nurse Fund in Denbigh, to which request a Guardian made the intelligent objection that "he was against voting the ratepayers' money for any purpose other than the relief of the poor." In reply Mr. Bircham said that the Board had power to subscribe to such an object, and "he thought that Boards of Guardians ought to help such institutions in every way possible, and not throw cold water on them on the mere technical quibble that the benefit of the nursing did not extend over the whole union. Those parishes that had shown sufficient intelligence and enlightenment to establish a nursing institution should be encouraged. He hoped soon to see Welsh-speaking nurses appointed throughout Wales." Mr.

Bircham further added that in his opinion the Guardians in the whole course of their duties could not find a better or more practical way of relieving the destitute poor than by granting financial aid for the maintenance of district nurses, and putting themselves thereby in a position to secure their services for the sick poor; a view which must be cordially endorsed by all sensible people.

### HELP FROM THE CHAPELS.

The universality of the interest taken by every portion of the community in the progress of district nursing is well illustrated by the many and various methods adopted in different localities to raise money for local institutions. At Hebden Bridge, Yorkshire, a musical performance takes place annually in aid of the funds of the Nursing Institution, "the *locale* of the performance being changed about yearly between Salem Wesleyan Chapel and Hope Baptist Chapel." This year the former was the scene of action, the principal feature of the programme being Sir Joseph Barnby's cantata, "Rebekah." The chapel was crowded, the congregation numbering nearly 1,100 people, and the collection amounted to the creditable sum of £19 6s.

### QUEEN'S NURSE AT STAMFORD.

The annual report of the Stamford branch of the Queen Victoria Jubilee Institute for Nurses is a satisfactory one. Nurse Blackmore has had a somewhat increased number of cases to deal with during the past year, and the "Queen's inspector's" comment on the "efficient and kindly manner" in which her duties have been discharged is cordially and unanimously endorsed by the branch committee. Financially, the society is free from debt, and has begun the present year with a small balance in hand. Amongst other kindly help received from friends, the committee gratefully acknowledge a useful gift of shirts, tickets for a convalescent home, and the use of a carriage.

### ST. MICHAEL'S CONVALESCENT HOME, WESTGATE.

A very largely-attended ball was given the other night by Lady Halsbury in aid of the Convalescent Home at Westgate-on-Sea, in which she has interested herself for many years. The Inner Temple Hall was placed at her disposal for the purpose, and the ball proved a triumphant success, and will doubtless result in some substantial financial help for the home. The building is not yet complete, a sum of £2,500 being required to finish the block which is to be devoted to men patients.

### FOOD, DRESSINGS, AND DIGNITY.

A nurse at Warwick has ventured to think that food and surgical dressings are not a pleasant combination in a dining-room; and the *Birmingham Mail* headed its report of her complaint, "Discontented Workhouse Nurses at Warwick." The Mayoress of Leamington suggested that another arrangement might be made, but the chairman considered that the dignity of the Board was at stake, and scorned such a compromise. He preferred to accept the resignation of the nurse in question, trusting no doubt to meet with a successor who would agree with him that a room which "had done for several years" would still continue to serve for other purposes as well as for a refectory. It is possible that the chairman of the Warwick Board may find it less easy than he anticipates to meet with a competent infirmary nurse willing to follow the practice of unlucky predecessors who probably feared to face the unjust accusation of being "discontented" if they demanded ordinary consideration.

### MEDICAL MISSIONS IN INDIA.

Encouraging reports of the work of the mission hospitals in India will be found in the current number of the *Zenana*. Miss Jenkins, L.R.C.P. and S., writes from the Lady Kinnaird Memorial Hospital at Lucknow, telling of the great need there for additional space, many serious cases having to be kept waiting for admission owing to this reason. It is hoped that this desired extension may be accomplished this year, and that building may be soon begun. "We try," goes on the writer, "as far as possible, to have the same routine and discipline here as in a small English hospital. It is not easy to teach our nurses habits of order and punctuality, and the same lessons have to be taught over and over again; but we feel nursing offers a most important career to young native Christian women, and we try to make their training as complete as possible." For the Duchess of Teck Hospital at Patna twenty-one cots have been provided for by friends in Scotland, being supported annually, some by single individuals, some by counties or towns, and others by various branches of the Young Women's Christian Association. One cot has been endowed "in remembrance of many mercies received at Patna during the Indian Mutiny."

### SHORT ITEMS.

Miss Julia Cock, M.D., has been elected as a member of the committee of management of the Royal Free Hospital, this being the first occasion upon which a woman has been associated with the management of the hospital.—H.R.H. the Princess Christian and Lady Jeune have resigned their connection with the Home of Rest for Nurses at Brighton.—The Queen has forwarded fifty pounds of cast linen to the Linen Rag Society for use in the Church Missionary Society's hospitals in India, China, Palestine, Egypt, and Persia.—A woman has just been appointed to the post of vaccination officer at Cheltenham. She is the daughter of one of the relieving officers of the Cheltenham Union.—A public dinner is to be held in Worcester after Easter in celebration of the one hundred and fiftieth anniversary of the opening of the infirmary. An appeal is then to be made for funds to build a nurses' home, increased and better accommodation for the staff being much needed.—Lady Wimborne, whose interest in hospital and nursing matters is well known, is just now trying to set on foot a plan to provide a trained district nurse for the three villages of Canford, Kinson, and Longham. The proposal should meet with cordial local support, for a nurse's services are much needed.—Nurse E. Williams, district nurse at Burley-in-Wharfedale, was the other day presented by public subscription with a set of books on nursing, a case of instruments, and a gold watch and chain.—The annual general meeting of the Slough Nursing Fund was held on February 28th.—Nurses may be glad to know that the Cosmopolitan Dress Cutting School have arranged evening classes on Tuesdays and Thursdays from six to nine at much reduced fees, at the Teaching Department, 65a, Oxford Street, W.

### Midwifery Papers.

### I.—THE FEMALE PELVIS.

In the practice of midwifery, it is necessary to have some knowledge (which must be accurate as far as it goes) of the female pelvis, its structure, contents, and functions. It is, indeed, exceedingly necessary for the midwife to be able to distinguish the presence of any causes which may complicate or, perhaps, prevent normal delivery, such as malformations of the pelvis or strange growths in any of the parts; and an acquaintance with the normal conditions will make it possible to detect the abnormal, and to summon in good time the necessary aid of the medical man. The pelvis is a bony ring made up of four bones; two ossa-innominata form its front and sides, and meet the sacrum or cross-bone, with its continuation the coccyx, which closes it in behind. Each os-innominatum is formed by the union of three bones (the ilium, ischium, and pubes), and spreads out like a wing on each side of the sacrum, then, curving inwards, meets in front and forms the pubic arch. The sacrum is a triangular wedge-shaped bone, articulating with the ilia on both sides, forming the sacro-iliac joints, and with the coccyx beneath forming the sacro-coccygeal joint, which is a movable hinge-joint, acting during labour.

In the adult woman the various articulations of the pelvic bones are provided with cartilage, fibrous structure, and some synovial membrane, which, during pregnancy, become swollen and softened; the bones become slightly separated, increasing the mobility, thereby lessening the danger of fracture from pressure induced by pregnancy and labour. The pelvis is internally divided by a bony ridge, called the ilio-pectineal line, which runs round the upper margin or inlet into the true and false pelvis. The false pelvis is that portion which is above the ilio-pectineal line; the true pelvis, which lies beneath, is more closely connected with our subject. The upper opening of the true pelvis is called the brim or inlet; the lower opening, the outlet; and the part lying between, the cavity. Diameters: The pelvic diameters are three at the brim, three in the cavity, and three at the outlet. They are the autero-postero (from front of pelvis to back), the transverse (from side to side), and the oblique (from a point right and left of sacrum to a point in front taken obliquely). The measurements are as follows:—

| | Autero-Posterior. | Transverse. | Oblique. |
|---|---|---|---|
| Brim | From 4 to 4½ in. | 5 to 5¼ in. | 4½ to 4¾ in. |
| Cavity | ,, 4½ to 4¾ in. | 4½ to 4¾ in. | 4¾ to 5 in. |
| Outlet | ,, 4¼ to 4½ in. | 4 to 4¼ in. | 4½ to 4¾ in. |

The diagonal conjugate is measured from the apex of the pubic arch to a point of the sacrum called its promontory, which can be felt in examination on or near the pectineal line. This diameter should be from 4½ to 4¾ inches; if it is found to measure under 4 inches great difficulty will be experienced in delivery, occasionally giving rise to great danger, and it will be better to send for a medical man early in the labour. The promontory of the sacrum can be felt only in a contracted pelvis, so a medical man's skill is necessary to assist the labour. The female pelvis is specially adapted in structure for its function of child-bearing. It is shallower, wider, and less funnel-shaped than the male pelvis; the pubic arch, and the inlet and outlet are all much wider, the promontory of the sacrum less projecting and the coccyx is movable; all of which facts conduce to the ease and security of the birth. The depth of the pelvic cavity and the curvature of the sacrum have an important influence also in labour; if the cavity is shallow and the sacral curvature moderate the labour is likely to be easy and natural, while, if the cavity is deep and the curvature correspondingly great, the labour is likely to be tedious and even difficult. The bones of the female pelvis are thinner and slighter and the muscle attachments less strongly marked than in the male. The true pelvic cavity is lined with muscles, and blood-vessels and nerves are distributed throughout, and are liable to injury from pressure and laceration during labour. The internal organs of generation are contained in the true pelvis, and are (1) the ovaries, in which the ova are formed; (2) the Fallopian tubes, the excretory ducts to the ovaries; (3) the uterus; (4) the vagina. The bladder and urethra, which lie in front of the uterus, and the rectum, lying behind, are also found in the true pelvis.

(1) The ovaries (about 1½ inches long) lie on either side of the uterus, and are supported between the folds of the broad ligament which is formed by the peritoneum. (2) The Fallopian tubes conduct the ova from the ovaries to the uterus, and are placed in the thickness of the broad ligament. They open at one end into the fundus (or broad end) of the uterus, and the other (the fimbriated extremity) lies close to the ovary to receive the ovum during menstruation. (3) The uterus is a hollow muscular organ lying in the pelvis between the bladder and the rectum, and above the vagina which helps to support it. It is also held in position by the broad and round ligaments. It is pear-shaped, and its upper part or broad end is called the fundus, the middle portion is called the body, and the lowest and narrowest end is named the cervix or neck, and terminates in a small opening called the os-uteri. The uterus is composed of strong muscle and fibrous tissue, and its cavity is lined by mucous membrane containing numerous glands. The length of the adult uterus is about 2 by 2¾ inches, and its weight is from 1 to 2 ounces. A fold of peritoneum which comes down behind the uterus, between it and the rectum, is called Douglas's pouch, in which are occasionally found small tumours, or abscesses, which at times interfere with labour by pressure. (4) The vagina is the canal of communication between the uterus and the vulva, and is made up of muscular and cellular tissue, it is lined internally by mucous membrane, which lies in thick folds along its surface. It is from 2½ to 3 inches long; the anterior wall being slightly longer than the posterior. The lips of the vulva enclose the external opening of the vagina, and also the urethral opening. The perinæum lies between the vaginal and the rectal openings, and is of great use in supporting the internal organs from below.

### Royal British Nurses' Association.

We have received the following notice from the Secretary:—

The next quarterly meeting of the General Council will be held on Friday, April 10th, 1896, at five p.m.

The fourth sessional lecture of the season will be given on Friday, March 20th, at eight p.m., at the offices of the Royal British Nurses' Association, 17, Old Cavendish Street, by Miss de Pledge, Lady Superintendent of the Chelsea Infirmary, the subject being "The Village of Palaces." Members are admitted free, the general public on payment of 1s.

### Appointments.

National Hospital for the Paralysed and Epileptic, Queen Square, Bloomsbury.—Miss Tweed, who has been acting as Lady Superintendent for the last seven months, and who has been attached to this hospital for some years, has been appointed Lady Superintendent vice Miss Fiennes Clinton, resigned.

Royal West of England Sanatorium, Weston-super-Mare.—Miss Ethel Mawe has been appointed Lady Superintendent of this institution. Miss Mawe has had wide nursing experience. She has held the post of sister at the Royal Hospital for Diseases of the Chest, City Road, and at the Civil Hospital, Gibraltar, besides having worked in New Zealand, Japan, and elsewhere, her last appointment being that of sister of surgical wards at the Egyptian Government Hospital, Cairo. Her testimonials are excellent. We cordially wish her all success in her new work.

### Royal National Pension Fund for Nurses.

The following report of the Council was presented to the ninth general meeting of the members, held at the offices, 28, Finsbury Pavement, London, on March 12th, 1896:—

The Council have the pleasure to submit to the members of the Fund their report, with the annexed accounts and balance-sheet, prepared in conformity with the statutory requirements, and made up for the period of twelve months ending December 31st, 1895.

PENSION BRANCH.—During the twelve months there have been received 915 proposals (including 11 brought forward from last account) for granting pensions for £16,337 2s., of which 100 proposals, for assuring the sum of £1,634 15s. 4d., are still under consideration or were not proceeded with; 822 policies were granted (7 being in substitution for policies previously existing, leaving 815 net) for assuring £82 of immediate annuities, and £14,665 6s. 8d. of deferred annuities; producing £8,884 0s. 7d. in single payments, and £9,550 15s. in annual premiums; 4,389 policies have been issued from the commencement of the fund.

SICKNESS BRANCH.—In this department there have been received 420 proposals (including eight brought forward from last account) for weekly sick pay assurance of £265 12s. 6d., of which 96 proposals for assuring weekly sick pay of £43 12s. 6d. are still under consideration or were not proceeded with; 325 policies were granted, securing £187 10s. weekly sick pay, producing £299 14s. in annual premiums; 1,409 policies have been issued from the commencement of the fund.

INVESTMENTS.—The investments on December 31st, 1895, amounted to £267,098 19s. 4d., as follows:—

| | Year 1895. | £ s. d. |
|---|---|---|
| In British Government securities ... ... ... ... ... | 31,133 7 11 | |
| In Colonial (Crown colonies) stock ... ... ... ... | 10,000 0 0 | |
| In India Government stock ... | 6,081 7 0 | |
| In railway and other debentures and debenture stocks | 115,117 14 6 | |
| Ditto shares (preference and guaranteed) ... ... ... | 16,583 18 10 | |
| In Foreign Government securities ... ... ... ... ... | 68,596 11 1 | |
| In municipal corporation bonds and stocks ... ... ... | 8,598 9 0 | |
| | | 256,111 8 4 |
| INVESTMENTS OF THE JUNIUS S. MORGAN BENEVOLENT FUND. | | |
| In railway and other debentures ... ... ... ... | £6,684 10 6 | |
| Ditto shares (preference and guaranteed) ... ... ... | 2,140 0 0 | |
| In Foreign Government securities ... ... ... ... ... | 1,105 0 6 | |
| | £9,929 11 0 | |
| Loans to policy-holders ... | 1,058 0 0 | |
| | | £10,987 11 0 |
| Total invested funds ... ... ... | | 267,098 19 4 |
| Cash balances, outstanding interest, &c. ... | | 5,448 11 6 |
| Total funds ... ... ... ... | | £272,547 10 10 |

PENSION BRANCH.—The growth in the amount of business done during 1895 is remarkable. On the pension side 915 proposals were dealt with during the year, as compared with 610 in 1894 and 533 in 1893; and 822 pension policies were issued, as against 563 in 1894 and 497 in 1893. To meet the wish expressed by many nurses, an arrangement has been made in recent years whereby premiums are received in advance, 2½ per cent. interest being allowed thereon. During 1895 £1,225 was received in such premiums, against £930 in 1894 and £703 in 1893. Every year this plan becomes more popular with the nurses, and thrift is thereby encouraged, for the interest gained is a great attraction. It is satisfactory to observe that the percentage of withdrawals tends to diminish rather than to increase, a proof of the prosperity of nurses generally. They have realised that the savings put by in the Pension Fund must be regarded as sacred, and the majority of nurses rightly consider these savings as absolutely beyond their reach for any other purpose than as a means of protecting themselves against age or other incapacity for work. It is interesting to find that nearly 50 per cent. of such withdrawals are due to the fact that the members are giving up nursing as a calling. The letters received from those who withdraw testify to the great assistance which the Fund has been to them by husbanding their savings against the hour of need.

SICKNESS ASSURANCE BRANCH.—The sick-pay policies are becoming more popular every year. Four hundred and twenty proposals have been dealt with in 1895, against 297 in 1894 and 260 in 1893. Three hundred and twenty-four policies have been issued, against 228 in 1894 and 216 in 1893. The sick pay which was disbursed was £909, as compared with £739 in 1894 and £576 in 1893; the number of nurses receiving these benefits being 177, as compared with 149 in 1894 and 129 in 1893.

EXPENSES OF MANAGEMENT.—By Bourne's Comparative System, one of the standard insurance handbooks, the expenses of management, which were 4·62 per cent. in 1893, fell to 4·32 in 1894 and 3·41 in 1895. This steady reduction in the cost of management is the more satisfactory seeing that the number of letters received during the year amounted to 24,745, an increase of 8,300 over the previous year; the letters despatched were 23,562, an increase of 6,320; whilst 12,324 separate receipts for premiums alone were issued; and 1,353 cheques were drawn for sick pay. In this connection it may be mentioned that the work of the Fund is causing the average nurse to gradually become a better business woman. Fourteen hundred nurses have visited the office during the year. Both personally and in their correspondence they have evinced an increasing knowledge of the principles of insurance, and a marked development of business habits.

THE WORK DONE BY THE FUND.—Some idea of the extent of the benefits conferred by the Fund will be gathered from the statement that upwards of £20,000 has been returned to nurses in cash; more than £3,000 has been distributed amongst the members in sick pay; and the pensions paid to nurses already amount to £2,363.

DONATION BONUS FUND.—The Council have great pleasure in recording the marked increase in the amount of the Donation Bonus Fund. No less a sum than £23,610 has been added to it during 1895 by the generosity of Mr. J. B. Robinson, Messrs. Wernher, Beit, and Co., Mr. W. H. Burns, Mr. J. Pierpont Morgan, Lord Iveagh, Messrs. Rothschild, Mr. Daniel Marks, Messrs. Marks, Bulteel, and Co., Mr. S. Neumann, Messrs. L. Hirsch and Co., Messrs. Ansell, Mankiewicz, and Tallerman, Mr. E. Cassel, Mr. Robert Gordon, and others; and the Council take this opportunity of publicly expressing their grateful thanks to the donors. It has always been the aim of the Council to accumulate a capital sum of £20,000 for every 1,000 nurses who enter the Fund until the Donation Bonus Fund amounts to at least £100,000, and this object has been materially furthered in the year under review. The Donation Bonus Fund now amounts to over £67,400, the whole of which has been invested.

THE JUNIUS S. MORGAN BENEVOLENT FUND.—An interesting report dealing with this branch of the work will be found on another page. In five years the Benevolent Fund has given away more than £1,400, a portion of which has been advanced to nurses in temporary distress, either by way of loans or to enable them to keep up their premiums until they were in a position to again provide the money out of their own resources. The Council are under considerable indebtedness to Lady Rothschild, the President, who has been indefatigable in her efforts to aid the nurses effectually. Always first at the meetings of the Fund, Lady Rothschild has proved an ideal president in every way. Cordial acknowledgment is made to the other ladies who constitute the Committee of Management, each of whom is most helpful. Special thanks are due to Miss Pritchard, the honorary secretary, and to Miss Leigh, the lady visitor, whose services are much appreciated by their colleagues on the committee as well as by the nurses.

RECEPTION AT MARLBOROUGH HOUSE.—On Friday, July 26th, 1895, after an interval of three years, H.R.H. the Princess of Wales, the President, presented, at Marlborough House, certificates—which had been specially designed by Mr. Arthur Hacker, A.R.A., to whom the thanks of the Council are due—to the third and fourth thousand nurses who have joined the Fund. Previous to the reception, Mrs.

Walter H. Burns, wife of the Chairman of the Council, and daughter of the late Mr. Junius S. Morgan, very kindly attended at the Queen's Hall and handed an invitation to each of the nurses which entitled her to be received at Marlborough House. The presentation of certificates passed off admirably, with perfect order. The kindness of T.R.H. the Prince and Princess of Wales, their family and household, will make the day memorable to all who had the honour of being present. These receptions bring home to the nurses a fact of which the Council are deeply sensible, that their President, H.R.H. the Princess of Wales, from the very outset, has taken a deep and genuine interest in the Fund, and that she is in the truest sense the leader and friend of its members, whom she regards as her nurses. Each of the nurses present wore the armlet approved by H.R.H. the Princess of Wales, which is made of silk in the Danish colours, and bears a shield on which is inscribed the coronet and monogram of H.R.H. the President.

Council and Officers.—It is with deep regret and sorrow that the Council have to report the lamented death of their colleague, Dr. Bristowe, F.R S., who had been a member of the Council since the institution of the Fund. Dr. Bristowe always displayed the keenest interest in the progress of the Fund, and was ever ready to promote the welfare of nurses by showing them how greatly to their advantage it was to join in at the commencement of their career. His fearless advocacy and defence of the Pension Fund at the commencement contributed materially to its popularity and success. At the time of his death the Council tendered to Mrs. Bristowe and to the family the expression of their sympathy with them in this heavy trial, and placed on record their appreciation of the services which Dr. Bristowe had rendered not only to the Royal Pension Fund for Nurses, but to the progress of nursing generally. The Council have to report with regret the retirement of Mr. Charles Cotes, owing to the pressure of other claims upon his time. To supply the vacancy created by the death of Dr. Bristowe, the Council have elected Dr. Herbert P. Hawkins, of St. Thomas's Hospital, who is also the treasurer of the Nurses' Co-operation. The Council have felt the importance of associating with the Fund the younger generation of those who have materially contributed to its success by their splendid liberality to the Donation Fund, and it is with genuine pleasure that they have to report that Mr. Walter S. M. Burns, Mr. Eric Hambro, and the Hon. Walter Rothschild have accepted seats upon the Council during the year.

Retiring Members.—The following five members of the Council retire from the Fund, all of whom being eligible offer themselves for re-election: Mr. Henry C. Burdett, the Hon. Herbert C. Gibbs, Dr. Herbert P. Hawkins, Mr. E. Murray Ind, and Mr. Arthur H. A. Morton.

Representatives of Policy-holders.—The articles of association provide that no more than eight representatives of the annuitants and policy-holders of the society may be added to the Council. The Council have therefore submitted the names of the following ladies, seven of whom were members of the Advisory Committee, and one of whom has been nominated by the nurses themselves. From this list the annuitants and policy-holders have been invited to select seven members to serve during the year 1896. Elected members during 1895: Miss C. Davidson, Brompton Hospital for Consumption, Brompton; Miss Mary L. E. Dunn, Q.V.J.I., Dublin; Miss E. Fisher, General Infirmary, Leeds; Miss L. M. Gordon, St. Thomas's Hospital; Miss K. H. Monk, King's College Hospital; Miss F. C. Nott-Bower, Guy's Hospital; Miss E. Vincent, St. Marylebone Infirmary. Nurses' nominee: Miss Mabel Cave, the London Hospital.

The Auditor.—The auditor, Mr. Frederick Whinney, of the firm of Messrs. Whinney, Smith, and Whinney, chartered accountants, retires in accordance with the articles of association, and, being eligible, offers himself for re-election.

The Staff and Offices.—Owing to the large increase in the work the Council have found it necessary to take the whole of the floor where their present offices are situated, so as to afford them the necessary accommodation. Despite the large increase in the business of the Fund, the percentage of cost of management shows a considerable diminution, as the figures already given prove. The Council have great pleasure in recording their sense of the zeal and devotion of the secretary, Mr. Louis H. M. Dick, and of his assistants, who have exerted themselves to the utmost to successfully overtake a volume of additional work which the progress of the Fund has entailed upon them.

### East London Nursing Society.

The annual meeting of the East London Nursing Society was held at the Mansion House on March 10th, the Right Hon. the Lord Mayor in the chair. The matrons and nurses belonging to the society were among the audience.

The Lord Mayor, in his opening remarks, stated that it gave him great pleasure to preside on this occasion. He thought it very fitting that the meeting should be held at the Mansion House, as the work of the East-end nurses was carried on in so many of the neighbouring parishes. He was pleased to note that "Assistant Ladies" formed a special feature of the society, and that they were the medium through which the poor received the food and clothing so necessary in cases of destitution and illness.

The Right Rev. the Bishop of Stepney spoke of the vast area of which he had spiritual charge, and of the demand of these poor districts for all the help that could be given. He considered that district nursing did something towards preventing the overcrowding of hospitals—as only 9 per cent. of the cases tended had to be sent in—and the transfer of the latter, when necessary, was materially facilitated by the nurses. He regretted the funds were not larger. The number of nurses required not only pointed out the existence of a great mass of misery, but also of a great deal of sympathetic relief. Unfortunately, the growth of the funds did not correspond with the increase of this still-growing society. Subscriptions were replaced by legacies owing to the death of friends, and by this means the society was a loser in course of time, and new subscribers were sorely needed. The Bishop proposed the adoption of the annual report and audited balance-sheet, and the re-election of the general and executive committee.

The motion was seconded by Mr. F. D. Mocatta, and carried. Mr. Mocatta said he was a great admirer of the society, which he had known intimately from its birth. He thought that everyone who enjoyed good health should do something to assuage the sufferings of the less fortunate. The surroundings of the poor were greatly improved by the ministration of the nurses, who introduced cleanliness and fresh air into fœtid, squalid attics, and were educational persons as well as tenders of the sick.

The Rev. Canon Elwyn proposed: "That this meeting acknowledges the value of the work done by the East London Nursing Society, and undertakes to make its claims more generally known." He said he had great pleasure in moving this resolution. He had just heard, with much regret, that illness prevented the Rev. Harry Jones, chairman of the society, from being with them. He had done much to nurse the society into its present position. Referring to the chairman's suggestion that the report should "be taken as read," he trusted that it would also be "taken away and read."

In seconding the motion proposed by Canon Elwyn, Dr. Oswald A. Browne spoke of the beneficent work of the nurses, and of the value of trained nursing to the poor. He alluded to hospitals as being not only schools of science, but schools of sympathy. It was the application of medical knowledge which formed the great charm of hospital work. The thirty-three nurses employed by the society had nursed 5,438 cases during the past year, and to these they had paid 130,483 visits. Kindly ministration to the dying was also a feature in their work, 570 patients having been nursed in their last hours. Dr. Oswald Browne thought very highly of the physical, moral, and spiritual value of a nurse's work. It was not now a question of extending the work of the society, but rather a matter for serious consideration as to whether its income would suffice much longer for keeping it firm as at present. He also urged the need for new annual subscribers.

The Treasurer explained that the society was no longer in debt, the £150 adverse balance having been removed by special donations.

The Rev. — Hare proposed, and Rev. J. Parry seconded, a vote of thanks to the Lord Mayor for presiding and for granting the use of the rooms for the meeting, and this was carried with enthusiasm.

### Private Nursing.

By Sister Grace.

### III.—PRIVATE NURSES' ATTITUDE TOWARDS THE DOCTOR.

Those engaged in private nursing necessarily meet with medical men who pursue very different forms of treatment in the same cases, and you must learn to accommodate yourselves easily to these frequent changes. This diversity of professional opinion increases the interest of the work, and is advantageous to the nurse, for you will assuredly gain something from each new experience. Remember the necessity of respectfulness of manner to the doctor in attendance, and endeavour to inspire the patient with absolute confidence in his skill. Invalids soon learn to confide greatly in their nurse, and to be dependent on her opinion upon almost all subjects; therefore you can see how serious it would be if you allowed the patient to see any doubt in your mind as to the advisability of some order from the doctor. Moreover, it is a very serious professional error on the part of a nurse, and suggests an untrained condition, to allow herself to question any treatment ordered by the doctor; you are there to carry out his orders conscientiously, not to criticise them.

If for any reason you think you have misunderstood him, or that he has not fully realised something you have told him, do not let your patient become aware of your thoughts, but seek an explanation with the doctor outside the sick room. It is impossible for a doctor to know all the changes which occur in a patient whom he sees only once a day, or, perhaps, only every few days. Try to give him any facts of importance, but do not enlarge upon them; it is for *him* to draw his own conclusions. It is advisable in all serious cases to keep careful notes of what nourishment the patient takes, and at what hours it is administered, and the same with medicines. By handing this to the doctor you have an opportunity of writing any statement on the paper of which you do not wish the invalid to become cognisant.

There is much more medical than surgical work in private nursing. Surgical cases are more rare, and many people who undergo operations prefer to do so as paying patients in hospitals or in surgical homes, so that you may feel sure that the majority of your cases will be medical. Heart disease in all its forms, diseases of the respiratory organs and of the kidneys, chiefly occupy the private nurse, diversified with a certain amount of typhoid nursing and infectious work of all kinds. You will also probably have some mental cases, where the sufferer is not exactly out of his mind, but when his mental state approaches perilously near to that condition. This is trying work for the nurse, and requires a great exercise of tact and patience and a marked faculty for observation. Then, again, you will have cases where the patient gives way to inebriety or has contracted a habit of drug taking. I know of no work that more heavily taxes the capabilities of a nurse than this; in some ways the cases are more difficult to manage than those where the patient's brain is actually disordered, because the victim of this weakness is often hardly an invalid, and therefore difficult always to have under control, and also because the nurse usually finds that she has some traitor in the camp. Manifestly stimulants or drugs must be brought in by some one, and it is a very difficult and invidious task to discover who that "some one" is.

You will, I feel sure, in common with almost all nurses, find the nursing of typhoid fever very interesting, one reason being that so much rests with the nurse. It is pre-eminently a disease that taxes her skill and patience, and for which mere physic can do but a little, a fact which doctors are quite ready to acknowledge. It must run its course, and all that can be done, speaking broadly, is to keep death at bay and try to assist nature in the work of cure. In times of war enteric fever is a fell camp follower, and the mortality from it is great, because we have not the necessary palliatives; pure water is usually unobtainable, and fresh milk, our great mainstay in typhoid fever, is more rare still, if not absolutely absent.

In private nursing you will find difficulties to encounter, in the shape and style of the patients' beds; nothing is more fixed and unalterable than the affection for the ancestral four-poster, and only those who have tried to nurse a helpless patient in one can form any idea of the difficulties of the situation. If by the exercise of tact you are enabled to effect a change, nothing is better than a small iron bedstead without a foot-piece, fitted with chain springs, and good hair mattress. Bed-rests are generally to be had, but if not a chair put across the bed will often answer the purpose, nicely covered, not to be unsightly. To counteract the distressing sensation of perpetual slipping down in the bed, which almost always oppresses those who are very weak (more especially those suffering from some forms of heart disease), and who are consequently more comfortable raised high at their shoulders, I have found a simple and effectual way to put a bolster under the thighs, and by means of webbing sewed at the two ends, fixing it firmly to the head of the bed.

You will soon discover that one of your troubles is to counteract the mistaken kindness of friends, who are anxious to contribute dainties to the patient's diet; very often this is of little consequence, but in those recovering from typhoid fever and gastric complications, it may be attended with fatal results. I wish to draw your attention to this particularly, because when you are nursing single-handed you are obliged to rely on the help of the friends, and during your rest time much harm may be done. In cases of typhoid fever especially, explain to them why no food must be given except in strict accordance with the doctor's orders, and why they must not allow the invalid to sit up or to turn in bed.

### Pleasant Quarters for Nurses.

(Contributed by a Hospital Matron.)

The approach of spring warns us that the holiday season will shortly be upon us again, with all its delightful anticipations and arrangements consequent on a well-earned vacation. Many of our readers are no doubt fortunate enough to possess pleasant homes and kind friends, but there are others whom providence has not so blest, and the question of where to go and how to go becomes one of anxious consideration. This applies especially to workers in fever hospitals, whose friends and relations, particularly if they have children, are not always anxious to welcome as a guest one who has so recently come from an atmosphere of infection. In a delightful village in one of the loveliest parts of Hertfordshire a charming home has been opened by Mrs. Osborne, who, being a nurse herself, understands the needs and requirements of the members of her profession. Here nurses are received for such periods as may be required at the moderate sum of 15s. per week, with separate bedroom inclusive, or in the case of two sharing a room at 12s. 6d. per week. That it may be a holiday in the true sense of the word, no hard and fast rules are laid down, and the tired nurse can, if she will, enjoy the luxury of breakfast in bed. The journey from Liverpool Street to Sawbridgeworth station, the village in which the house is situated, can be accomplished for something under 2s. 6d., which is reasonable enough, and very convenient for London nurses. Every advantage of country life is offered in this sylvan retreat, including fresh milk, butter, and new-laid eggs. For any further information we would refer our readers to Mrs. Osborne, The Limes, Sawbridgeworth, Herts.

### Training School Registries.

A paper read at the Superintendents' Convention, Philadelphia, Feb. 12th, 1896, by Louise Darche, Superintendent New York City and City Hospital Male Training Schools, Blackwell's Island, New York.

### I.—SCHOOL AND PROTECTIVE.

By training school registries I mean those registries for nurses—whether managed on the purely co-operative basis, or by school management alone—which have for their primary object the well-being and employment of the graduate nurse, in contradistinction to those registries or directories which make the necessities of the graduate nurse a means for furnishing a lucrative business for some individual or society.

The former registry extends its privileges equally to a limited number of duly qualified members, and at a nominal cost; the latter extends its membership indiscriminately, and in its efforts to obtain the largest monetary returns in membership fees, breaks down the barrier which should exist between the duly qualified nurse, the partially qualified nurse, and the aspirant who is not qualified at all.

It is not surprising that this system of registration should in time crowd out the better qualified nurse from the general directory, and we now find the best nurses seeking for some other plan by which they may obtain recognition and employment. This is especially true in cities where the general directory for nurses has held sway from the beginning, and where the school registry has not yet been introduced.

The best physicians in these cities are already finding out that the general directory, as now managed, is not to be depended upon for the best nurses; and they, too, are dissatisfied, and would be willing to patronise another plan.

This brings us to the point of how best to establish a registry for nurses which shall limit the membership, protect the interests of the community for which it is organised, guarantee good nurses to those who seek them, and represent to the public the school or schools from which its members graduate.

This close corporation protective registry for nurses may be organised in three ways: First, as a training school registry conducted as a part of the school management; second, as a co-operative school registry managed by a committee composed of the superintendent of the school and assistant officers and graduate nurses; third, as the purely co-operative registry, composed of and managed by the graduates themselves.

In speaking of the first-mentioned registry, I immediately recall to mind two very well managed registries, viz., the Bellevue School Registry and the Illinois School Registry. Both are controlled as a part of the school management. In the Bellevue Registry the superintendent of the school is applied to direct when a nurse is wanted. The managers engage a resident messenger boy, whose duty it is to notify the nurses, wherever resident in the city, of calls as transmitted to them by the superintendent. The superintendent's office is also the registry office, and the managers hold themselves responsible for the efficient work and conduct of their graduate nurses.

The Illinois Training School Registry is managed on the same principle, though it has become so large, and its graduates are so numerous, that an agent—one of the graduates of the school—is engaged by the school managers for this part of the school work. Whether called agent, or fourth assistant, I do not know; her office adjoins that of the superintendent in the hospital, and she can refer to the superintendent's judgment as occasion requires.

In a strictly school registry none but graduates of the school can become members, and the managers assume all responsibility. In contrast to English methods, the nurse receives all the money she earns, and the remuneration due her is paid herself personally at the expiration of the term of service in the family in which she has been engaged.

The few rules which are necessary for protecting the nurse in private practice, and which define her obligations to the families which shall employ her, and also her obligations to the registry, are formulated by the school managers. Thus the same fostering care and managing power which trained and educated the nurse in the school, now, through the school registry, protects her interests and looks after her welfare when she has graduated and works for herself outside.

The second kind of protective registry I have mentioned may properly be called the co-operative school registry. It is a make-shift between the school registry managed by the school, and the co-operative registry managed by the graduates. It is one which may be organised when the managers of a school do not take enough interest in their graduates to undertake the trouble and expense entailed by the management of a school registry, or when the graduates do not harmonise in sufficient strength to manage one for themselves.

Our own school, placed under exactly these conditions, began a co-operative school registry some five years ago, in which both the officers of the school and an equal number of graduates form a managing committee. This registry has proved a success, and for those who contemplate such an organisation I would refer them for a detailed account to the last December number of the *Trained Nurse*.

Undoubtedly, the most-easy-to-manage protective registry is the school registry pure and simple, and next to that the graduates' co-operative registry.

### The Trained Nurse.—An American View.

Referring to Mr. Malcolm Morris' article, entitled "The Monstrous Regiment of Nurses," published in the *Practitioner*, the *New York Medical Record* states: He cites some very striking illustrations of the presumption and magnification of the English trained nurse. American physicians have also seen something of this seamy side. We do not know that the particular troubles described by Mr. Morris are the predominant ones here. What most physicians do see is that the average trained nurse often develops into a trained gossip of the most lugubrious and exaggerated type. A large majority of them seem to think that, after their strictly technical work is done, the proper thing is to sit down and tell the patients about all the cases they have ever seen or nursed in their lives. As a result, the convalescent listener is left with her mind full of extraordinary pathological tales that have an unpleasant, lasting, and pernicious effect upon her mental make-up. The trained nurse of to-day, also, has so exalted her office that she is very frequently unwilling to do anything but attend to purely technical work. The idea of touching a duster or of attending to the care of the room, or of doing anything but pouring out medicine, putting on the proper applications, and taking temperatures in the most ladylike way, becomes revolting to the majority of them, so that one often sighs for the old-fashioned nurse, who would settle down and do everything for 10 dols. a week. There seems, indeed, to be rather a need, not only of the trained nurse, but of a modest and half-trained nurse, who will make fewer pretensions, charge less money, talk less about her profession, and attend more strictly to business.

### Wants and Workers.

"Enquirer" would be glad to hear of any homes, "private or otherwise," where hysterical patients are specially treated.

### Doctors and Nurses at Cork.

If we are to believe reports, which, however, we feel inclined to accept only with considerable modifications, the "battle of the clubs" at Cork has entered on a new and somewhat curious phase in consequence of the nursing institutes in that city having thrown themselves into the contest.

The good people of Cork seem to have a fondness for obtaining their medical attendance on what may be called co-operative principles, and all sorts of people, who can quite well afford to pay ordinary medical fees, have joined societies and clubs founded for the benefit of working people, for the sake of getting the services of doctors at rates which were only intended as a semi-charity for the poor. This proceeding the doctors have greatly resented, and some time ago, as the clubs refused to alter their rules as to the admission of members, in such a way as to limit their scope to the working classes, the doctors struck and refused to have anything more to do with them. The clubs, however, straightway imported some new men, with whom, naturally, the old doctors have not been on terms of the sincerest amity. It was, in fact, war to the knife, only the knife took the form of the boycott, and the imported doctors were severely let alone. A little time ago, however, the suspicion arose in the minds of various people who had had trouble in obtaining nurses from the various nursing institutes, that the reason of their difficulty was to be found in the fact that they were being attended by the boycotted medical men—that in other words the nursing institutes were siding with the established doctors against the new comers. This made admirable copy for the newspapers, and the excitement grew fast and furious. Nevertheless, even according to their own accounts, there seemed but small ground for the assertion that a conspiracy was on foot. Dr. Hobart, chairman of the Cork branch of the British Medical Association, tells the *Cork Constitutional*, that he was not aware of any action having been taken by the Association in the matter. Dr. Lee, the secretary, said that nothing had come under his notice in connection with the matter, no one seemed able to trace the origin of the mystery, and at a general meeting of the medical profession it was resolved to take no notice of the "libellous and unfounded charge."

Still there are ways of doing business in Ireland that are "quite Oirish, you know," and although there may have been no direct resolution passed or orders given, it is quite possible that the managers of the nursing institutes may have known pretty well what would be pleasing to certain doctors of position in the city. In fact, while from a letter from the Mayor of Cork, published in the *Cork Constitutional*, it seems that the lady superintendent of one of the institutes confessed that she had acted "in consequence of rumours and hints which had reached her indirectly from some of the medical profession," the Mayor also said "I am glad to be able to say, in justice to the doctors of Cork, that she was not able to state that anyone had influenced her directly in this matter."

Now as to the position which nurses should take up. It is perfectly clear that nursing institutes are in a sense commercial undertakings, and that as a mere question of business it is open to their managers to select what cases they choose or to refuse what cases they do not choose to attend. It may be perfectly good policy to side actively with the established doctors;—it may be bad. That is a purely local question, and, no doubt, hinges on the views the managers take as to where good business lies. So far, also, as the nurses in question are concerned they are bound to the institutes they serve and must go only where they are sent. There need be no difficulty about that.

But it must be distinctly laid down that nurses in general are in no way tied by this action of the doctors. Medical men may quarrel or make peace, they may combine, may boycott, or may play the part of blacklegs, all this is nothing to nurses, who are in no way called upon to take sides. The doctors on both sides are qualified men, and there is nothing derogatory in nursing for any of them. It is a mere question of policy, and where angry passions rise to the height they evidently do in Cork it is easy to believe that a nurse will not improve her prospects by going against the majority of the doctors. Still that has to be decided by institutions rather than by nurses, these have merely to obey superintendents; and as for independent nurses, they are free to do exactly as they like and take the consequences, as also the institutes will have to do.

We may hazard a suspicion, however, that, after what has happened, if the clubs should perchance beat the doctors, they will not be long in "sweating" the nurses also, and introducing a system of co-operative nursing of their own. It is by no means likely that well-to-do tradesmen who want to have their medical attendance for less than a penny a week will long put up with paying a couple of guineas a week and her keep for the services of a nurse! The interests of the nurses as a whole are the same as those of the doctors who protest against organisations which have been arranged for the benefit of the poor being misused for the advantage of the well-to-do.

### Bournville—A Cocoa Colony.

A remarkable little colony is springing up around Messrs. Cadbury's huge cocoa factories at Bournville, in Worcestershire (near Birmingham), having its origin in the cordial relations which exist there between employers and employed. Some 1,400 women alone find work here, and the rate of pay is from 1s. 6d. to 2s. per week higher than in any other similar factory in the world, while the comforts and advantages are most unusual—indeed, almost unique. The working hours are seven and a half daily (with Saturday's half-day, making an average of under seven hours), and the works are shut for a fortnight's holiday every year. The factories open at 8.50 in the morning, and close at 5.30. An hour is allowed for the middle day meal, which is eaten in the large dining halls, one with seating room for 1,600 girls, another for 600 men, and a third for 70 clerks. Material for dinner can be brought by the workers in the morning, and cooked free of charge. There is also a supplementary refreshment bar at which food can be obtained at a very low tariff, such as plates of beef, ham, &c., at 1d., hot fruit pies 1d., three-quarters of a pint of tea or coffee ½d., glass of milk ½d., and so forth. The firm defrays the entire working expenses of the dining-rooms and large kitchens, and the milk and fruit comes from their farms. There are charming gardens laid out for the use of the workers.

A trained nurse is employed and paid by the firm, who attends to every case of sickness, a little consulting room and office being allotted to her in a cottage close to the main entrance. A sick fund provides weekly payments in cases of illness, and also in cases where members are compelled to absent themselves on account of infectious illness in their families. There is a special house for the use of those girls who have no homes, or whose parents live too far away to allow of daily journeyings to and fro. Another and larger home is about to be provided for this purpose.

But the new and particular feature of the colony is this. The Bournville estate (exclusive of the factory and its surrounding 60 acres) consists of about 140 acres, which are now to be laid out with the object of "making it easy for working men to own houses with large gardens" secure from interference by building with sun, light, and air; this land will be let on lease for 999 years at a ground rent of a halfpenny to a penny per yard, and houses will have to conform to a given size. A piece of ground will be given by the owners for purposes of recreation, and for the erection of schools, baths, and institute, which will be under a committee appointed by the tenants. The lines upon which this large undertaking is to be carried through are said to be most practical and business-like, and Messrs. Cadbury welcome inspection of the estate, and are glad to explain the nature of the facilities offered to settlers; and these, by the way, need not necessarily be connected with the factories. They are open to all.

The spirit of co-operation is largely developed amongst Messrs. Cadbury's employés and tenants, as the number of societies, technical schools, clubs, and special funds which have sprung into existence testify.

The factories are close to Bournville Station, which is reached from Birmingham by a constant service of trains.

### Everybody's Opinion.

[Correspondence on all subjects is invited, but we cannot in any way be responsible for the opinions expressed by our correspondents. No communications can be entertained if the name and address of the correspondent is not given, or unless one side of the paper only be written on.]

### WORKHOUSE INFIRMARY NURSING.

"A NURSE ON THE SOUTH COAST" writes: I have read with pleasure the remarks in THE HOSPITAL about workhouse infirmary nursing, and feel how gratifying it is that there is someone to sympathise with nurses whose work is most trying and disheartening at times. I have been under the Poor Law for some years now, but have had previous experience in hospital routine. Few outsiders realise what the responsibility of the work is in a country infirmary or convalescent home, where supervision of the patients is often so difficult. A good nurse thinks nothing too much or too hard in the way of her duty or the comfort of her patients, whether paupers or otherwise; but I do find the majority hard to please, difficult to keep in order, and the general arrangements very bad.

### HELPERS OR RIVALS?

"A READER" writes: In an article published in THE HOSPITAL" of February 22nd, viz., "Helpers or Rivals? The Nurse Question," we find ourselves very unkindly spoken of. We do not wish to give vent to a volley of accusations, as does the compiler of this article, but wish merely to protest against an unfair attack on the profession generally merely because a few individual women have assumed a wise air at the bedsides of their patients and disputed the doctor's diagnosis. Has it been done? Admitted a "little learning" is a very dangerous thing, hence we find a new probationer, who has done a few months' hospital work, much more ready to air an opinion than a fully-trained nurse would be. It is generally understood amongst us—in fact, one of the first principles taught—that to give an opinion to a patient concerning their ailments is extremely "bad form," whatever one's thoughts may be. The amount of study and "learning" required to become an efficient nurse would not be likely to disturb any woman's "mental balance," were that "balance" as weak as our antagonist would make out. Women having proved the extent of their mental capacity, there is no need to discuss the "brain power" question. An "egotistical and self-conscious" nurse would be unbearable; but how often do we meet her? All these undesirable little qualities are "nipped in the bud" in our first year's probabation, that is if we show any signs of developing the same! I have worked often with the much-pitied "general practitioner," and have not had any misunderstanding or unpleasant little episodes such as are mentioned, neither do I consider him a man to be despised—rather the reverse. Of course, we "obey" the doctor in every detail; but is not the question of "honour" rather unique? Quite as new an idea as the "changing of medicines and prescriptions" by a nurse. Much would depend on the "man"; it would not be his "practice" we should honour! Why this attack? Is the object of it to crush the female and extol the male sex?

### LOCKERBIE DISTRICT NURSING ASSOCIATION.

MRS. ALEX. ROGERSON, joint hon. secretary Lockerbie District Nursing Association, writes: A copy of THE HOSPITAL "Nursing Mirror" of February 1st, 1896, was sent to me, and as it contains a notice on "Working Hours at Lockerbie," in which our committee and lady superintendents are severely censured, I laid it before the said committee at our quarterly meeting yesterday, and was requested by those present to send you a copy of our "rules," by which you will see that the "humane policy" which is advised in your article is what we endeavour to carry out, though the exigencies of the work occasionally make our nurse feel that it is quite impossible to adhere strictly to hours. She told me she had a hearty laugh over the article in question, as she knows how very anxious the committee are that she should not overtax her strength in any way, although in the cases mentioned it had been quite unavoidable. We had, in our annual report, dwelt specially upon her unselfish devotion to her work, as we wished to emphasise the difference between her and her predecessor, who had left a most unfavourable impression on the public mind. I had yesterday to read a letter to the committee from Nurse Hadley, expressing her thanks for all the encouragement and consideration she had received. I trust this explanation will be satisfactory.

[We fail to see that this "explanation" in any way betters the facts to which exception was taken in the "Nursing Mirror" of February 1st. That Nurse Hadley is in the habit at times of pressure of being on duty for many hours in excess of those dictated by humanity and common sense is not denied in the letter we publish above, and, whatever the cause may be, such a condition of affairs is reprehensible in the highest degree. It is of little use to have a printed rule to the effect that "the nurse shall devote eight hours a day to visiting and ministering to the sick poor" if in practice "the exigencies of the work," which in district nursing are likely to be frequent, are allowed to stretch the working day of eight hours into one of fourteen or twenty. Many a nurse will, if unrestricted, labour beyond her strength with "unselfish devotion to her work," but this very fact increases, rather than diminishes the responsibility incurred by those who continue to allow her thus to sacrifice her own health and future powers of usefulness.—ED. *T.H.*]

### Notes and Queries.

### Queries.

(181) *District Nursing.*—Will you tell me if, at the age of twenty-two, I am too old to be trained as a district nurse; and, if not, where should I apply to get the required training?—*Minnie.*

(182) *Maternity Home.*—Will you tell me if there is a maternity home at any seaside place near London?—*Nurse G.*

(183) *Obstetrical Nursing.*—Will you recommend me any work on the treatment of cases of puerperal fever?—*Nurse.*

(184) *Lady Roberts' Nurses.*—Where can I get information about Lady Roberts' Nursing Institute in India?—*India.*

(185) *Maternity.*—Please tell me if there is any maternity hospital in London which would take a young married lady as paying patient, and what would be the charge?—*Nurse Rose.*

(186) *Technical Instruction.*—Can you tell me if there are any particular lectures on nursing used for the above, what they are, and where they can be obtained? Also if the lecturer must have passed any particular examination?—*A Constant Reader.*

(187) *Co-operation.*—Can you give me any information as to the amount of percentage paid by nurses belonging to provincial co-operative institutions?—*C. E. A.*

(188) *Sea Voyage.*—I am ordered a sea voyage after a severe breakdown in health from one's work, but cannot afford it unless I can find a patient to go with, or take a post as stewardess. Can you advise me how to set about obtaining one or the other?—*Matron.*

### Answers.

*An Irish Nurse, Elspeth, Trained Nurse,* and *A Constant Reader* enclose neither name nor address. As stated above, no attention can be paid to anonymous queries.

*Platt* has also omitted name and address, and encloses postal order for 4s. If this writer will supply the missing information the postal order shall be returned and the query replied to in this column, for which no fee is required.

(181) *District Nursing (Minnie).*—Before you would be eligible for district nursing you must have had at least a year's training in some general hospital or infirmary, and probationers are not usually received at such institutions under twenty-five. You might obtain admission to a children's hospital as a preliminary step if you have really made up your mind to nursing as a profession and do not wish to lose time. You should read "How to Become a Nurse" (Scientific Press, 428, Strand, W.C.), in which you will find full information as to the best steps to take with regard to training.

(182) *Maternity Home (Nurse G.)*—There is a maternity hospital at Brighton. You will find a list of homes and hospitals in Burdett's "Hospital Annual" (Scientific Press, 428, Strand, W.C.).

(186) *Technical Instruction (A Constant Reader).*—Write to Miss Lamport, 52, St. John's Wood Road, for information on lecturing.

(187) *Co-operation (C. E. A.).*—Each institution makes its own rules and fixes the percentage paid by the nurses. Most careful inquiries should be made by nurses before entering upon an engagement, for it sometimes happens that such institutions are co-operative only in name.

(188) *Sea Voyage (Matron).*—Watch the advertisement columns in the London daily papers, and in THE HOSPITAL, and advertise yourself. Invalids going abroad frequently need the services of a nurse, and you are quite likely to find a patient if you are not tied to any special route. For information with regard to an appointment as stewardess you should apply to the offices of the large shipping companies, whose addresses you will find in a London 'Post Office Directory, or in the daily papers. We hope you will be successful in finding what you want, and that a voyage will completely restore your health.

### For Reading to the Sick.

### MERCY.

**Motto.**

Mercy carried infinite degrees
Beyond the tenderness of human hearts!
—*Wordsworth.*

**Verses.**

Of all the precious gifts, O Lord,
Thy mercy can impart,
Whate'er Thou willest to withhold,
O grant a perfect heart. —*William Bright.*

New every morning is the love
Our wakening and uprising prove;
Through sleep and darkness safely brought,
Restored to life and power and thought.

New mercies each returning day
Hover around us while we pray;
New perils past, new sins forgiven,
New thoughts of God, new hopes of heaven.

If on our daily course our mind
Be set to hallow all we find,
New treasures still, of countless price,
God will provide for sacrifice. —*Keble.*

When all Thy mercies, O my God,
My rising soul surveys,
Transported with the view, I'm lost
In wonder, love, and praise. —*Addison.*

The quality of mercy is not strained;
It droppeth as the gentle rain from heaven
Upon the place beneath! it is twice blessed—
It blesseth him that gives, and him that takes!
'Tis mightiest in the mightiest! It becomes
The thronèd monarch better than his crown:
His sceptre shows the force of temporal power,
The attribute to awe and majesty,
Wherein doth sit the dread and fear of kings;
But mercy is above this sceptred sway,—
It is enthronèd in the hearts of kings,
It is an attribute of God Himself! —*Shakespeare.*

**Reading.**

"With great mercies will I gather thee. In a little wrath I hid My face from thee for a moment; but with everlasting kindness will I have mercy on thee, saith the Lord thy Redeemer."—*Isaiah* vii. 8.

I can seldom say more to God for those I love, than: "Do as Thou hast ever done; be to them as Thou hast been to me; be Thou their portion in time and in eternity!" I rarely can enter into particulars in prayer, but have more facility in returning thanks for special mercies. It has been well said that, "He who records God's mercies will never want mercies to record."—*M. S.*

"And now I sit alone in this silent room, and I think on the mercies of my God to me. My strength has been, I now see, that sense of entire dependence upon Him, which kept me, as it were, clinging to the arm which upheld me." —"*Words of Thankfulness.*"

God's mercy lies everywhere. Whenever we see Christ, He is imparting blessings as the sun imparts light and warmth. While He was here on the earth, He was always reaching out His hand to give a benediction to some life that sorely needed it. Now it was on the children's heads, now on the leper, now on the blind eyes, now on the sick, now on the dead, that He laid those gracious hands, and always He left some rich gift of blessing. . . Then it is a striking fact that the last glimpses we have of the Saviour in this world shows Him in the attitude of blessing.—*Rev. J. R. Miller.*

### Presentation.

National Hospital for Paralysed and Epileptic, Queen Square, W.C.—On March 5th Sister Cross was presented by the medical and nursing staff with a handsome gold watch and clasp, as a "token of respect and esteem," on her departure from the hospital to take up new work at the Plaistow Fever Hospital.

### The Book World for Women and Nurses.

[We invite Correspondence, Criticism, Enquiries, and Notes on Books likely to interest Women and Nurses. Address, Editor, The Hospital (Nurses' Book World), 428, Strand, W.C.]

The Tender Mercies of the Good. Christabel Coleridge. (Isbister and Co.)

Miss Coleridge stands apart from the "short story" compiling world in her latest contribution to literature. This is neither short nor sad—the two essential qualifications of the modern novelette. Of course, the authoress has a very definite design in her present work; though, were it not that the title initiates one somewhat, it might be difficult to discover it. The plot of the story is simple enough, but the telling of it is complicated. Miss Coleridge, we venture to surmise, draws her characters from the life, as the setting, a pastoral one, has a great sense of reality about it also. Austin Fairford is the hero; the story is of his fall and rise in life—that is to say that he is introduced to us first at the period immediately following his fall. Towards the conclusion of the book Austin sums up his record to a fellow sufferer in these words: "You and I, Jack, as you probably know, have had a very similar sort of record. I am aware that you know, indeed, you cast it up to me, that I came home, nearly six years ago, because I was in disgrace. You, too, I think, had got into trouble. Well, we had a fresh start, and our friends kept our secrets; in different ways, perhaps, but pretty much from the same motives. I don't know how you felt, but I had very little notion of how to pick myself up again, and I made a fool of myself in various ways, as you also know." . . . "Jack could not appreciate the force of the motive which made a young English gentleman so give away reserve and distance as to put his experiences in the power of one at once so near and so removed from him as the bailiff's son. . . . But it did in a degree come home to him that his companion did not stand up above him on a point of vantage unfairly gained, but was beside him in the gulf, bringing to him whatever power it was that had rescued him." Miss Coleridge shows a dramatic power and a close analysis in dealing with these two characters, round whose misdeeds the chief interest of the book centres.

### MINOR NOTICES.

No. 2 of "The Commonwealth," a social magazine, has been brought out, and, in spite of certain very leading characteristics of the new venture, we commend it to the reading public. The articles for the February issue are of an excellent literary value, containing "The Political Situation," by Canon Scott-Holland; "Emerson," by the Rev. A. L. Lilley, M.A.; and "A Prayer in Christ's Name," by Canon Gore. There is a frontispiece reproduced from one of Ford Madox Brown's picture in the National Gallery. "The Commonwealth" is to be obtained of Messrs. Smith and Sons, and the price monthly is 3d.

The "British Phrenological Year-Book," the first of a contemplated long-continued series of annuals, is before us for review. The Association of British Phrenology has chosen 1896 advisedly for their "Year Book," this being the centenary of Dr. Gall's first public declarations of his discoveries. The aim of the society, through the medium of their organ, is the indication of the progress which this particular branch of knowledge is making, "and to present to the general reader information which will interest and instruct." The book is excellently got up, both as to reading matter and illustrations, and to the thoughtful reader will present much material for reflection. Messrs. Nicolls and Co., 23, Oxford Street, are publishing the volume at 1s.

Mr. T. Fisher Unwin has published another neat little volume on "Good Reading about Many Books, Mostly by their Authors," the second year's reading (for 1896) being issued consequent on the success of the first volume of a like kind. A design of Joseph Pennell and portraits of all artists whose works are commended as "good reading" are given as illustrations.

